# Mapping the landscape and future directions of stem cell therapy for inflammatory bowel disease

**DOI:** 10.1186/s13287-026-04999-2

**Published:** 2026-04-16

**Authors:** Junyan Gao, Yilong Liu, Tian Xia, Zixuan He, Yu Bai

**Affiliations:** 1 Department of Gastroenterology, Changhai Hospital, Naval Medical University, 168 Changhai Road, Shanghai, China; 2 Department of Gastroenterology, Changzheng Hospital, Naval Medical University, Shanghai, China; 3 Changhai Clinical Research Unit, Changhai Hospital, Naval Medical University, Shanghai, China

**Keywords:** Stem cell therapy, Inflammatory bowel disease, Clinical landscape

## Abstract

This study systematically analyzed 170 registered clinical trials to clarify the global development landscape of stem cell therapy for inflammatory bowel disease (IBD) and its core challenges, and put forward corresponding future research directions. The results showed that the research on this therapy has accelerated significantly in the past three decades, with research geographically clustered in the US, China and Spain, and severe underrepresentation of African countries. The therapy is mainly focused on Crohn’s disease (CD), especially refractory perianal fistulas, and mesenchymal stem cells (MSCs) account for over 70% of the applied cell types. Among 88 completed trials, 29.5% achieved primary endpoints, showing preliminary efficacy, yet the field faces prominent challenges: 20.6% of trials were prematurely terminated, early-phase studies dominate, Phase IV trials and long-term efficacy data are scarce, primary endpoints are highly heterogeneous, pediatric research is limited, and most completed trials have unknown outcomes. To address these issues, this study proposes that the future research should prioritize international cooperation to promote research equity, standardize efficacy endpoints, integrate long-term follow-up into study designs, optimize stem cell source and administration protocols, and implement precise patient selection. Overcoming these bottlenecks is essential to establish stem cell therapy as an effective complementary treatment for refractory IBD and improve the prognosis of patients unresponsive to conventional therapies.

**Supplementary Information:**

The online version contains supplementary material available at 10.1186/s13287-026-04999-2.

Crohn’s disease (CD) and ulcerative colitis (UC), representing the two principal subtypes of inflammatory bowel disease (IBD), are chronic progressive disorders characterized by relapsing-remitting courses [1], with a global prevalence exceeding 0.3% [2]. The therapeutic paradigm of stem cell transplantation for IBD traces its origins to serendipitous clinical observations in patients with IBD complicated by hematologic malignancies. A landmark study by Drakos et al. in 1993 documented the first evidence of CD remission following autologous hematopoietic stem cell transplantation in a patient with concurrent non-Hodgkin’s lymphoma and IBD [3]. Subsequently, both autologous and allogeneic stem cell transplantation have evolved from opportunistic applications in comorbid conditions to targeted therapeutic strategies for refractory IBD patients with multiple comorbidities [4]. Nevertheless, comprehensive global clinical data encompassing temporal trends, geographical distribution patterns, and other critical dimensions in this field remains scarce. Through structured analysis of online clinical trial databases, we have mapped the historical and current landscape of stem cell-based interventions for IBD, providing detailed characterization of these trials along with discussions on research priorities and future directions. Our research is compliant with the TITAN Guidelines 2025 - governing declaration and use of AI [5].

We retrieved clinical trials investigating stem cell therapies for IBD from the Trialtrove database, using a search strategy that included the terms (Drug Type: cell type, stem cell) AND (Disease: Crohn’s Disease OR Ulcerative Colitis). As of April 18, 2025, 155 trials were initially identified, and 153 were retained after manual exclusion of two irrelevant records. We further cross-referenced our findings with ClinicalTrials.gov, identifying 17 additional trials not captured in the Trialtrove database, resulting in a final count of 170 trials.

Stem cell therapy for IBD has undergone transformative development over the past three decades, evolving from bench research to clinical translation. Global clinical trial analysis reveals 170 registered studies, with 88 completed (51.7%), 28 currently active (16.5%), and 11 in protocol design phase (6.5%) (Fig. 1A, Supplementary Table 1). Our analysis of the 88 completed trials indicates a total enrollment of more than 3,600 participants, providing a substantial baseline for evaluating the feasibility and preliminary safety of these interventions. The research trajectory demonstrates remarkable acceleration: from an initial cohort of 11 trials in 2004–2007, the number surged to 47 between 2020 and 2023, with 16 new trials launched in the past two years (Fig. 1B). Developmental phase distribution shows predominance of early-stage exploration, with 121 phase I-II trials (71.2%), compared to 16 confirmatory phase III trials (9.4%) and 29 phase IV studies (17.1%), indicating most therapeutic approaches remain in preliminary efficacy validation stages (Fig. 1B, Supplementary Table 1).

**Fig. 1:**
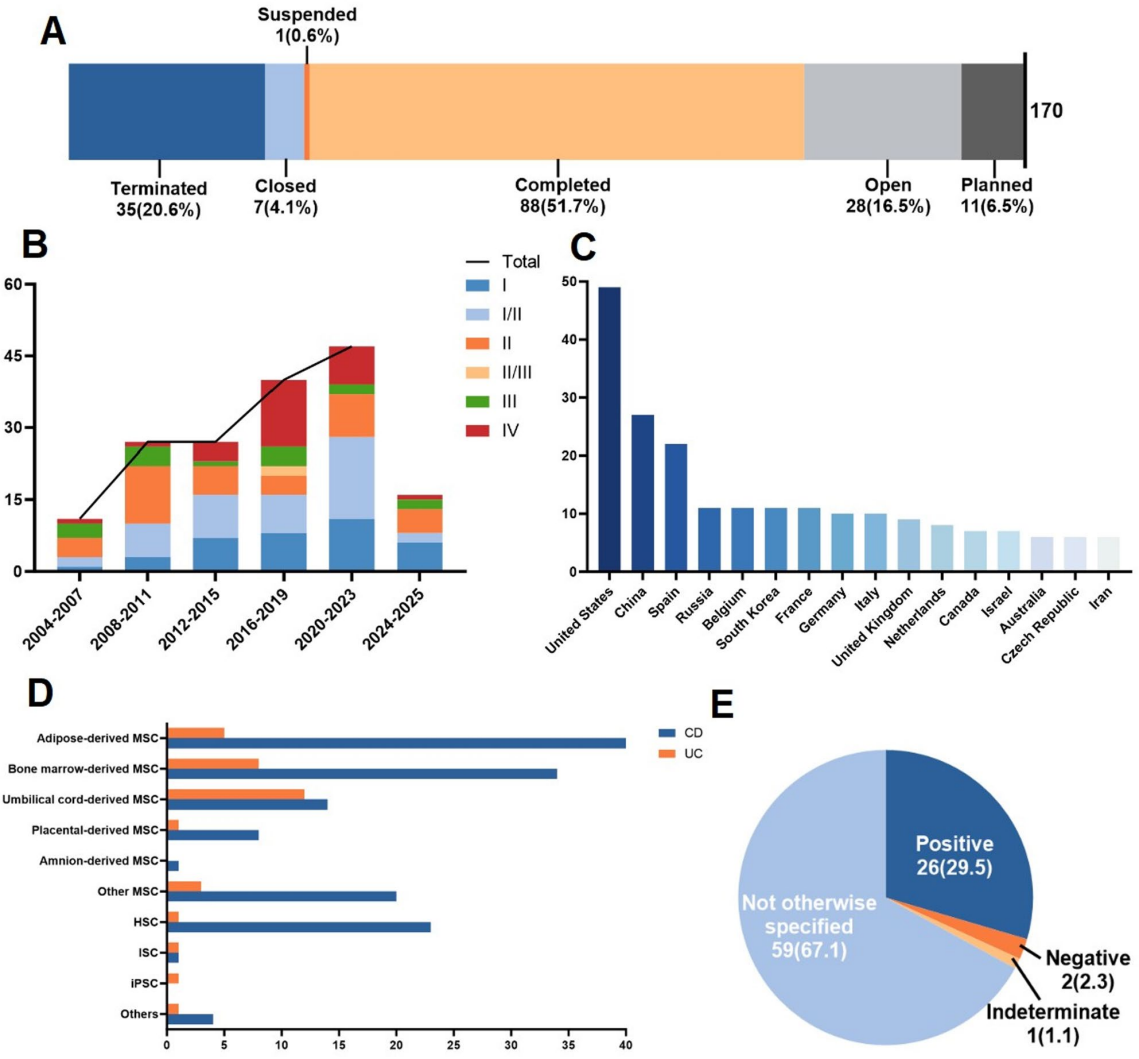
Global landscape of stem cell therapy trials for IBD (*n* = 170). **A**: Stem cell therapy trials, by status, as of April,17 2025. **B**: Clinical trial volumes categorized by four-year intervals from 2004 to 2025, including independent analysis for 2024–2025 data. Each interval displays total trial counts and phase I-IV distributions. **C**: Number of clinical trials in the top 16 countries and regions. **D**: Quantitative mapping of clinical trials by stem cell therapeutic strategies and disease classifications. *MSCs*: mesenchymal stem cells, *HSCs*: hematopoietic stem cells, *ISCs*: intestinal stem cells, *iPSCs*: induced pluripotent stem cells. Others include adipose-derived stem cells, human amnion epithelial cells, etc. **E**: Efficacy analysis of completed clinical trials

Beyond the temporal trends, the global landscape of IBD stem cell research exhibits distinct geographical clustering and marked regional disparities (Fig. 1C, Supplementary Table 2). These disparities are partly associated with the distribution of IBD incidence rates and disease burden. Furthermore, differences in national regulatory frameworks for stem cell therapies and variations in clinical trial registration practices may also contribute to the observed geographical imbalances. The United States maintains a commanding global lead with nearly 50 clinical trials, accounting for 28.8% of the total research portfolio in this therapeutic domain. Among the top 16 participating countries, five are from the Asian region. China has become the regional leader with 27 studies (15.9%). Although China is still a low-incidence area for IBD overall, the incidence and prevalence of IBD have been rising rapidly in recent years [6]. Spain spearheads European contributions with 22 trials (12.9%). However, Morocco is the only African contributor in this research field, highlighting the systemic inequality in research capacity and participation. The relatively low incidence rate of IBD in the African region, combined with limited medical resources, may be one of the reasons for its low participation in stem cell research. Beyond disparities in clinical research infrastructure and funding, the pathogenesis of IBD is closely linked to industrialization, lifestyle, and westernized dietary patterns. Its relatively lower incidence in socioeconomically underdeveloped regions may, to some extent, influence the perceived urgency and resource allocation for related clinical research there. Funding analysis reveals academic institutions driving 107 studies (59.8%), complemented by 57 industry-sponsored trials (31.9%) (Supplementary Table 1). The presented data unveil systemic developmental challenges confronting stem cell therapy in IBD research: critical limiting factors including imbalanced global allocation of scientific resources, deficient specialized funding in specific nations, and underdeveloped medical research infrastructure, which collectively and significantly hamper the progression of cutting-edge clinical studies.

Additionally, Analysis of 170 clinical trials reveals significant disease-specific focus: 146 studies (85.9%) target CD, predominantly refractory perianal fistulas, versus 32 trials (18.8%) for UC. Notably, the analysis of administration routes showed a clear preference for local injection (e.g., perianal or intralesional) in CD trials targeting fistulas, while systemic intravenous infusion was more commonly employed in trials for luminal CD and UC, highlighting how the route of delivery is tailored to the disease phenotype. Therapeutically, mesenchymal stem cells (MSCs) therapies dominate, with umbilical cord-derived (e.g., TH-SC01), bone marrow-derived (e.g., Remestemcel-L), and adipose-derived (e.g., Darvadstrocel) MSCs collectively constituting over 70% of interventions (Fig. 1D, Supplementary Table 3). Paradigm-shifting innovation emerges from Japan’s intestinal epithelial stem cell transplantation trial (jRCTb032190207), which pioneers endoscopic mucosal transplantation for in situ tissue repair, establishing a novel paradigm for organoid-based regenerative therapy. Mechanistically, Stem cell therapy exerts therapeutic effects on IBD through multifaceted mechanisms, including modulating T cell activity, promoting the release of anti-inflammatory factors, regulating immune responses, and repairing the damaged intestinal mucosal barrier [7]. This comprehensive approach directly targets chronic intestinal inflammation at its pathological core. Efficacy outcomes demonstrate that among 88 completed trials, 26 (29.5%) met primary endpoints (positive results), whereas 2 (2.3%) showed endpoint failure (negative results) (Fig. 1E, Supplementary Table 1). Nevertheless, a critical issue is that a substantial proportion of completed trials (67.1%) remain with unknown or uncertain outcomes. Among the clearly reported outcomes, the case of Cx601 is particularly instructive. While the European ADMIRE-CD Phase 3 trial (NCT01541579) [8] of Cx601 (darvadstrocel) met its primary endpoint of combined fistula remission at week 24, the subsequent US-led ADMIRE-CD II trial (NCT03279081), using an identical intervention, primary endpoint, and protocol, failed to demonstrate statistical superiority over placebo, despite enrolling a smaller number of participants. This geographic discrepancy highlights potential differences in patient populations, fistula preconditioning standards, or disease characteristics across regions, as full US results continue to be analyzed. Together, these trials serve as crucial “negative signals”, revealing core challenges in translating regenerative medicine into clinical practice. Furthermore, the high proportion of completed trials in public clinical trial registries that either did not report or failed to update primary outcome data has created a significant evidence gap. Many of these are small early-phase studies, limited by underpowered designs, lack of publication incentives, or methodological shortcomings, which constrain interpretability and contribute to publication bias. In contrast, the above well-designed randomized controlled trials, despite their negative results, exemplify scientific integrity through complete and transparent reporting. Another major challenge is the significant heterogeneity in primary endpoints across different trials, which greatly complicates cross-study comparisons and meta-analyses. Currently, few high-quality meta-analyses specifically focusing on stem cell therapies for IBD have been published, and most are limited by marked heterogeneity in primary endpoints across included trials, which further highlights the urgency of global endpoint standardization for this field. For example, trial endpoints for luminal CD range from clinical remission scores to endoscopic improvement, while fistulizing disease primarily focuses on fistula closure assessed through imaging or clinical evaluation. Although such variations reflect the complex pathophysiology of IBD, they pose significant obstacles to evidence synthesis and efficacy determination. The analysis of heterogeneity in this study can assist in adjusting analytical strategies, uncovering sources of variation, and enhancing the reliability of conclusions when conducting high-quality meta-analyses in the future. At the clinical translation level, these findings may provide a basis for understanding the differential effects of various stem cell therapies across specific populations, dosages, and other subgroups, thereby advancing precision clinical applications. Additionally, they can guide future research directions and promote the design of targeted randomized controlled trials. It is important to note that this registry-based analysis reports trial success as defined by primary endpoints; it does not allow for the assessment of effect sizes or direct comparative efficacy across different stem cell therapies.

Despite the increasing number of clinical trials exploring stem cell therapy for IBD, numerous challenges remain. First, 20.6% of trials have been prematurely terminated due to recruitment difficulties or funding interruptions, highlighting persistent feasibility and sustainability challenges in the clinical translation of stem cell therapy for IBD. Second, our analysis identified that only 17 of the 170 trials (10.0%) included pediatric patients (under 18 years old). Among these 17 pediatric trials, 7 have been successfully completed, 5 were terminated, and 4 are ongoing. This limited representation underscores the ethical and practical challenges of conducting stem cell therapy research in children, where such interventions often await initial safety and efficacy data from adult populations. Third, a significant gap in evidence has emerged: only 17.1% of studies have progressed to the IV phase of monitoring, and there is an urgent need for large-scale real-world evidence and longitudinal follow-up cohorts to validate the durability of treatment.

Based on existing problems, the future prospects of stem cell therapy for IBD need in-depth exploration in several key areas, with the core being strengthening international cooperation, promoting research equity, and optimizing therapy-related technologies and protocols. In terms of international cooperation, first, international funding agencies and major research consortia can establish special funding programs to prioritize support for stem cell clinical research in low- and middle-income countries. Second, promote the construction of regional shared research platforms and core laboratories. Concentrate resources to establish international standard-compliant centers for cell production, quality control, and data management, providing technical support for neighboring countries and lowering the entry threshold for research teams. Third, encourage and standardize the open sharing of clinical data. Under regulatory and ethical frameworks, facilitate cross-border access to high-quality research data for secondary analysis, enabling researchers in resource-constrained regions to conduct rigorous evidence-based research and gradually improve their scientific research capabilities. In terms of technology and protocol optimization, it is necessary to identify the optimal source of stem cells, optimize extraction and culture methods to ensure their quality and safety; at the same time, determine the appropriate administration route, dosage, and treatment frequency, and accurately screen the patient population most likely to benefit.

In summary, although stem cell therapy for IBD is in a dynamic development stage with increasing global participation, its clinical translation still faces prominent obstacles such as high dropout rates and the dominance of early-stage research. Addressing these identified challenges—optimizing cell products, clarifying standardized treatment protocols, and accumulating robust long-term evidence—is crucial for the development of this field. If these bottlenecks can be successfully overcome, stem cell therapy has significant potential to develop into a clinically validated adjunctive strategy for selected patients with refractory disease, thereby improving the prognosis of many patients with refractory IBD.

Provenance and peer review.

Not commissioned, externally peer reviewed.

## Supplementary Information

Below is the link to the electronic supplementary material.

Supplementary Material 1.

## Data Availability

Not applicable.
