# Supplementary material for: Mapping the landscape and future directions of stem cell therapy for inflammatory bowel disease
Source: Stem Cell Res Ther. 2026 Apr 16;17:141. doi: 10.1186/s13287-026-04999-2 (PMC13088503; doi:10.1186/s13287-026-04999-2)
Supplement: Supplementary file 1 — Supplementary Material 1. [file 13287_2026_4999_MOESM1_ESM.docx]

Supplementary table 1. Descriptive characteristics of clinical trials that use stem cells for IBD

| **Characteristics** | **Trials, No. (%)** |
| --- | --- |
| Trial Status |  |
| Terminated | 35(20.6) |
| Closed | 7(4.1) |
| Suspended | 1(0.6) |
| Completed | 88(51.7) |
| Open | 28(16.5) |
| Planned | 11(6.5) |
| Trial phase^†^ |  |
| I | 36(21.2) |
| I/II | 45(26.5) |
| II | 40(23.5) |
| II/III | 2(1.2) |
| III | 16(9.4) |
| IV | 29 (17.1) |
| Sponsor type^*^ |  |
| Academic | 107(59.8) |
| Industry | 57(31.9) |
| Government | 9(5.0) |
| Cooperative Group | 2(1.1) |
| Miscellaneous | 4(2.2) |
| Outcomes of completed trials |  |
| Positive | 26(29.5) |
| Negative | 2(2.3) |
| Indeterminate | 1(1.1) |
| Not otherwise specified | 59(67.1) |

† 2 out of the 170 trials are pilot trials with no defined trial phase.

* Each of the 170 trials may include multiple types of sponsors

Supplementary table 2. Number of clinical trials in the top 16 countries and regions.

| **Countries and regions** | **Number** |
| --- | --- |
| United States | 49 |
| China | 27 |
| Spain | 22 |
| Russia | 11 |
| Belgium | 11 |
| South Korea | 11 |
| France | 11 |
| Germany | 10 |
| Italy | 10 |
| United Kingdom | 9 |
| Netherlands | 8 |
| Canada | 7 |
| Israel | 7 |
| Australia | 6 |
| Czech Republic | 6 |
| Iran | 6 |

Supplementary table 3. Quantitative mapping of clinical trials by stem cell therapeutic strategies and disease classifications.

| **IBD subtypes** | | **CD** | **UC** |
| --- | --- | --- | --- |
| **MSC** | Adipose-derived | 42 | 5 |
|  | Bone marrow-derived | 34 | 8 |
|  | Umbilical cord-derived | 14 | 12 |
|  | Placental-derived | 8 | 1 |
|  | Amnion-derived | 1 | 0 |
|  | Other | 20 | 3 |
|  | Total | 119 | 29 |
| **HSC** | | 23 | 1 |
| **ISC** | | 1 | 1 |
| **iPSC** | | 0 | 1 |
| **Others** | | 4 | 1 |
| **Total** | | 147 | 33 |

MSC: mesenchymal stem cells, HSC: hematopoietic stem cells, ISC: intestinal stem cells, iPSC: induced pluripotent stem cell. “Other” under the MSC category refers to mesenchymal stem cells used in the study that are either not specifically designated by tissue origin or have a broad source. The separate “Others” category refers to other cell therapy products that do not fall under MSC, HSC, ISC, or iPSC, primarily including umbilical cord blood mononuclear cells, amniotic epithelial cells, stromal vascular fraction, etc. Among the 170 studies, 10 included patients with both Crohn’s disease and ulcerative colitis.
